# Association of Alcohol and Tobacco Consumption with Depression Severity in the Oldest Old. Results from the Age Different Old Age Cohort Platform

**DOI:** 10.3390/ijerph18157959

**Published:** 2021-07-28

**Authors:** Janine Quittschalle, Alexander Pabst, Margrit Löbner, Melanie Luppa, Kathrin Heser, Michael Wagner, Hendrik van den Bussche, André Hajek, Hans-Helmut König, Birgitt Wiese, Matthias C. Angermeyer, Wolfgang Maier, Martin Scherer, Steffi G. Riedel-Heller

**Affiliations:** 1 Institute of Social Medicine, Occupational Health and Public Health (ISAP), University of Leipzig, 04103 Leipzig, Germany; Alexander.Pabst@medizin.uni-leipzig.de (A.P.); Margrit.Loebner@medizin.uni-leipzig.de (M.L.); melanie.luppa@medizin.uni-leipzig.de (M.L.); Steffi.Riedel-Heller@medizin.uni-leipzig.de (S.G.R.-H.); 2 Department of Neurodegenerative Diseases and Geriatric Psychiatry, University Hospital Bonn, 53127 Bonn, Germany; Kathrin.Heser@ukbonn.de (K.H.); Michael.Wagner@dzne.de (M.W.); 3 German Center for Neurodegenerative Diseases (DZNE), 53127 Bonn, Germany; Wolfgang.Maier@ukbonn.de; 4 Department of Primary Medical Care, Center for Psychosocial Medicine, University Medical Center Hamburg-Eppendorf, 20246 Hamburg, Germany; bussche@uke.de (H.v.d.B.); m.scherer@uke.de (M.S.); 5 Department of Health Economics and Health Services Research, Hamburg Center for Health Economics, University Medical Center Hamburg-Eppendorf, 20246 Hamburg, Germany; a.hajek@uke.de (A.H.); h.koenig@uke.de (H.-H.K.); 6 Institute for General Practice, Hannover Medical School, 30625 Hannover, Germany; wiese.birgitt@mh-hannover.de; 7 Center for Public Mental Health, 3482 Gösing am Wagram, Austria; angermeyer@aon.at; 8 Dipartimento di Sanità Pubblica, Università degli Studi di Cagliari, 09124 Cagliari, Italy; 9 Department of Psychiatry and Psychotherapy, University Hospital Bonn, 53127 Bonn, Germany

**Keywords:** depression, lifestyle factors, old age, gender differences

## Abstract

This study aimed to examine the association of alcohol and tobacco use with severity of depression in older age. Analyses were performed on a pooled data set (n = 3724) from two German old-age cohort studies (LEILA 75+, 6 follow-ups and AgeCoDe/AgeQualiDe, 9 follow-ups). Depressive symptoms were assessed via two screening scales for depression (CES-D and GDS-15) which were harmonized for pooled analysis. A mixed-effects linear regression model for the total sample and additional stratified models for men and women were used. Smoking at baseline was significantly associated with a higher level of depression severity (β = 0.142, 95% CI: 0.051–0.233, *p* = 0.002), whereas drinking was significantly associated with a decreased level of depression (β = −0.069, 95% CI: −0.119–−0.021, *p* = 0.005). Concurrent substance use at baseline increased longitudinal depression severity (β = 0.193, 95% CI: 0.011–0.375, *p* = 0.037). Analyses stratified by gender showed a significant inverse association between drinking and depressive symptoms in men (β = −0.138, 95% CI: −0.231–−0.045, *p* = 0.004), but not in women (β = −0.060, 95% CI: −0.120–0.001, *p* = 0.052). Given the burden of major depression, it is important that health care providers, especially primary care physicians, assess and monitor lifestyle factors, even at older ages.

## 1. Introduction

Depression is a common condition in old age [1,2]. The prevalence of depressive disorders in people aged 75 years and older is 17.1% and 7.2% for major depression [3]. Depression is often associated with a multitude of negative consequences including reduced quality of life, increased suicide rate [2], and functional impairment [4]. In addition, it has been shown that depression in later life can occur as a prodromal or risk factor for later dementia [5] and appears to have a poorer prognosis compared to younger age groups [6], making the older and oldest old particularly vulnerable age groups.

Additionally, depressive symptoms in late life are associated with public health consequences. Research has revealed that the average annual direct health care costs of depressed older adults exceed the costs for non-depressed individuals by almost one-third [7]. Information regarding risk and related factors are therefore necessary to understand and provide insight on demographic trends. Risk factors which are associated with severity of depressive symptoms include various biological (e.g., gender) and psychosocial factors (e.g., life events, lifestyle) [8,9]. Lifestyle factors, such as drinking and smoking, are of particular importance because they are modifiable. Drinking and smoking are common in the Western world [10,11]; they are also leading risk factors for a variety of diseases, impairments and mortality [10,12,13].

With regard to the risk of late life depression, very few studies have examined the association between substance consumption behavior and the risk of late life depression [14,15,16]. While there is evidence that smoking is associated with severity of depression in older individuals [15,17,18,19], research findings are inconsistent regarding alcohol consumption. A number of studies found no association between depressive symptoms and alcohol consumption [14,15,20,21,22,23], while some researchers found an inverse association between alcohol use and depression severity [24,25]. However, substance use is often not limited to one substance. Previous research shows a strong association between drinking and smoking: heavy alcohol users are therefore more likely to be heavy smokers, and smoking appears to be associated with later heavy drinking [10,13,26]. This is also true for the oldest age group, where a relationship between smoking and drinking has also been found [26,27,28,29]. This concurrent pattern of use is gaining importance because the combined use of alcohol and tobacco poses an increased risk for physical and mental health disorders and greatly increases mortality [13,28,30,31]. In addition, there is evidence that women and men exhibit different patterns of use. For example, older men appear to be at higher risk for using several substances concurrently and in a hazardous way [29,30,32].

Previous research that has investigated risk factors for depression in the oldest old while considering lifestyle factors [14,15,21] has neglected the effect of concurrent drinking and smoking behavior. To our knowledge, no study has explicitly examined the concurrent use of alcohol and tobacco and their association with severity of late-life depression. Thus, the present study attempts to fill this research gap. Greater knowledge of risk factors for late life depression may help to identify at-risk groups among the older and oldest old in order to provide appropriate interventions. Therefore, the aim of the present study is to determine the association between current drinking and smoking and severity of depressive symptoms in late life, particularly among those aged 75 years and older, with a special focus on concurrent use and gender differences.

The following research questions will be examined:

1. Is there an association between substance use (drinking, smoking and concurrent use) and depression severity?
2. Does this association between substance use (drinking, smoking and concurrent use) and depression severity vary by gender?

## 2. Materials and Methods

### 2.1. Study Design and Sample

Data were derived from two prospective German old-age cohorts: the “Leipzig Longitudinal Study of the Aged” (LEILA 75+) and the “German Study on Aging, Cognition, and Dementia in Primary Care Patients” (AgeCoDe/AgeQualiDe) as part of the project “Healthy Aging: Gender Specific Trajectories into Latest Life (AgeDifferent.de)”. More detailed information on both studies has been published elsewhere (e.g., AgeCoDe: Luck et al., 2007 [33]; LEILA 75+: Riedel-Heller et al., 2001 [34]). Both studies were conducted between 1997 and 2016 and recruited participants who were at least 75 years of age at baseline (AgeCoDe: primary care patients in six German cities (Bonn, Düsseldorf, Hamburg, Leipzig, Mannheim, Munich); LEILA 75+: general population via local registry office plus a small portion of institutionalized subjects in Leipzig).

The datasets were pooled to increase the sample size and the stability of the model’s estimates. Overall, there were 5019 participants in the pooled dataset of the AgeDifferent.de platform at baseline (n = 1692 from LEILA 75+, n = 3327 from Age-CoDe/AgeQualiDe). Participants from the original cohort studies provided written informed consent prior to study participation. The local ethics committees approved both studies.

**Figure 1:**
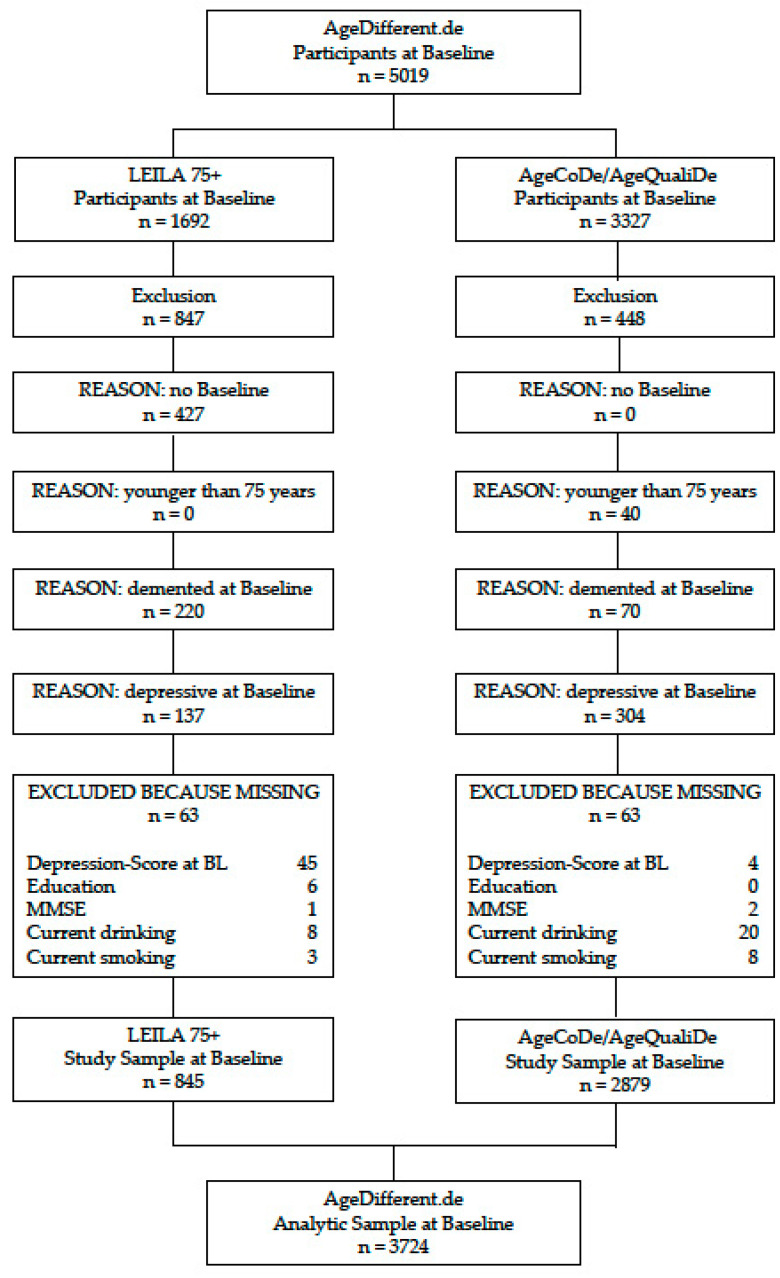
Flowchart of the sample.

The present study includes data on covariates and predictors assessed at baseline and information on depression severity up to the last follow-up of both studies (AgeCoDe: follow-up 9) observation period of up to more than 13 years; LEILA 75+: follow-up 6 (observation period of up to more than 16 years). Of the overall sample, 427 (8.51%) participants did not complete the baseline assessment and another 40 (0.8%) participants were younger than 75 years of age and therefore excluded. We further excluded 290 (5.78%) participants with a diagnosis of dementia at baseline, as well as 441 (8.79%) participants with a diagnosis of depression at baseline, identified by accepted cut-off values for the CES-D and for the GDS-15 (reported in detail below). Further, participants with missing data in depression score and relevant covariates at baseline were excluded (n = 97, 1.94%). The final analytical sample consisted of 3724 participants; a flow chart is depicted in Figure 1.

### 2.2. Measures

#### 2.2.1. Depression

The severity of depressive symptoms was the primary target variable and was assessed by two scales. In LEILA 75+, the depressive symptomatology was assessed using the German version of the depression scale from the Center for Epidemiologic Studies Depression Scale (CES-D) [35,36], which is a self-report depression scale for research use in the general population. In AgeCoDe/AgeQualiDe, the short form of the Geriatric Depression Scale (GDS-15) was used [37]. The CES-D contains 20 items with answers ranging from 0 (rarely or not at all) to 3 (mostly). The CES-D has good sensitivity (88%), but slightly lower specificity (73%) [38]. In order to identify depressive symptoms at baseline, we used a cut-off score of 22/23 (non-case/case) [39]. The GDS-15 is used to screen for depression in later life [40]. This measurement is comprised of 15 yes/no questions. In a systematic review, Pocklington et al. [41] found a sensitivity of 0.89 and a specificity of 0.77 for the GDS-15 at the recommended cut-off score of 5/6 (non-case/case). The dichotomized depressive scores based on established cut-off values for the CES-D and for the GDS-15 were used only to exclude participants with depression at baseline. For further analyses, the discrete sum scores were used.

In order to combine depression scores from both studies, it was necessary to develop a harmonized depression measure based on the CES-D and GDS-15 scales following the approach by Lipniki et al. [42], and based on Griffith et al. [43]. In line with this approach, we first normalized scores using Blom’s method [44]. Second, we winsorized scores with greater or lower than three standard deviations around the arithmetic mean. Lastly, we standardized depression scores by converting to z-scores within each study using estimated means at common values (i.e., averages from data pooled across the two studies), obtained from regression models adjusting for age, gender, education and all corresponding interactions.

#### 2.2.2. Drinking

Alcohol consumption at baseline was assessed as the self-reported number of days with alcohol consumption per week within the last 12 months (LEILA75+) or currently (AgeCoDe/AgeQualiDe). Answers were dichotomized to form a measure of the prevalence of drinking, indicating the consumption of alcohol on at least one day per week (coded 1) versus less frequently (coded 0).

#### 2.2.3. Smoking

The smoking status at baseline was assessed in both studies by categorizing current smokers, ex-smokers and non-smokers. Smoking referred to cigarettes in LEILA 75+ and additionally included cigars and pipes in AgeCoDe/AgeQualiDe. Smoking behavior was dichotomized to form a measure for smoking prevalence that indicated current use (coded 1) compared to former or never-use of tobacco products (coded 0).

#### 2.2.4. Concurrent Substance Use

Concurrent substance use was classified as follows: (1) yes (0) no. The category “yes” refers to concurrent drinking and smoking as defined above, but not necessarily simultaneous use. The category “no” refers to no substance use or single substance use only (alcohol or tobacco).

#### 2.2.5. Covariates

The study from which data originated was included as a covariate in all regression models in order to control for any unobserved heterogeneity between both original cohort studies. Additional variables were assessed as follows: Gender (women or men) and age (in years) were assessed at baseline. Marital status was categorized as single/divorced, married, and widowed. Education was measured by the highest completed level of education and was recoded into low, medium, and high according to the revised version of the international new CASMIN educational classification [45]. As the detection of depressive symptoms may be affected by cognitive impairment [3,46], the Mini-Mental State Examination (MMSE; [47]) was included as a control variable. The MMSE score ranges from 0 (worst score) to 30 (best score).

### 2.3. Statistical Analyses

All analyses were performed using Stata 16.1 SE (StataCorp LP, CollegeStation, TX, USA). Level of significance was set to α < 0.05. Descriptive results are presented as mean ± SD or absolute frequencies and percentages. In order to examine factors associated with severity of late-life depression on average, we constructed mixed-effects linear regression models with the harmonized depression score as the outcome variable. Four different models were run. In the first step, drinking and smoking prevalence (model 1) and an additional interaction term between drinking and smoking prevalence (model 2) were entered and adjusted for covariates (gender, age, education, marital status, MMSE, study). In a second step, the analyses were stratified by gender (model 3 = women, model 4 = men), and models were adjusted for all covariates reported above, except for gender.

## 3. Results

### 3.1. Participant Characteristics

Socio-demographic characteristics at baseline of the entire study sample and separated by gender are presented in Table 1. The participants’ average age was 80.1 years (SD = 3.9), with a range of 75 to 99 years. Two-thirds of the sample were woman (2469/3724, 66.3%) and the majority was low educated (2454/3724, 65.9%). Almost half of the participants (1750/3724, 47%) were widowed; of female participants 60% (1482/2469) were widowed.

### 3.2. Prevalence of Substance Use

Table 2 shows the substance use at baseline of the entire study sample and separated by gender. Half of the sample reported drinking alcohol at least once a week (1875/3724, 50.4%). Most participants did not currently smoke (3472/3724, 93.2%). Hardly any of the participants reported concurrent use of both substances (140/3724, 3.8%). Subsequent descriptive analyses in the pooled sample revealed several significant gender differences at baseline. Women were slightly more likely not to smoke (*p* = 0.013). Men, also, tended to drink more often (*p* < 0.001). In addition, men showed significantly more often concurrent use (*p* < 0.001).

### 3.3. Association between Substance Use and Depression Severity

To assess the association of drinking and smoking with severity of depression on average, mixed-effects linear regression models were conducted (see Table 3). Results revealed that smoking was significantly associated with higher levels of depressive symptoms (β = 0.142, *p* = 0.002). Drinking was significantly associated with a lower level for depression severity (β = −0.069, *p* = 0.005) (Model 1). In addition, an interaction effect was found. The concurrent use of alcohol and tobacco significantly increased the expected level of depression severity above and beyond the individual risks (β = 0.193, *p* = 0.037) (Model 2).

### 3.4. Gender-Specific Associations between Substance Use and Depressive Symptoms

**Figure 2:**
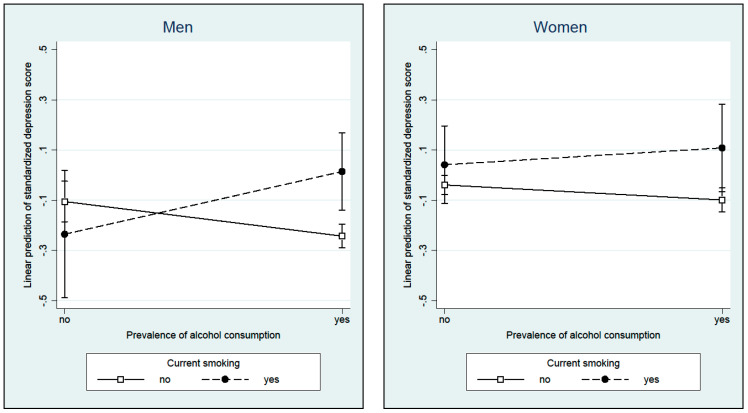
Gender differences for substance use.

Table 4 presents results of the stratified models analyzing gender differences in the association of drinking and/or smoking with severity of depression on average. In women (Model 3), no significant effects in terms of depressive symptomatology were found between those who smoke and drink (β = 0.127, *p* = 0.301) as well as those who only smoke (β = 0.081, *p* = 0.321) or only drink (β = −0.060, *p* = 0.052). In men (model 4), the interaction effect of concurrent substance use was significantly associated with higher levels of depression severity (β = 0.388; *p* = 0.013), whereas those who only drink without smoking showed significantly lower levels (β = −0.138, *p* = 0.004). Figure 2 displays the gender differences regarding concurrent substance use.

## 4. Discussion

The present study used national data from two pooled German old-age cohorts of adults 75 years and older to examine the association between lifestyle factors such as drinking and smoking and depression severity in late life. Moreover, this study is the first to present data on the association of concurrent drinking and smoking with severity of late-life depression on average. In general, our results revealed that smoking is associated with higher levels of depression symptoms. Furthermore, we found an inverse association between alcohol use and depression severity. According to gender-stratified analyses, the inverse association between drinking and depressive symptoms was found only in men. In addition, the concurrent use of both substances was associated with subsequent higher depression severity. This association persisted after adjustment for covariates (sex, age, education, marital status, cognitive impairment, study).

Our findings are consistent with previous research linking smoking to poor physical and mental health [15,18,19,48,49,50,51,52,53]. Specifically, it has been shown that both current and former smokers are at increased risk for depression [31]. In addition, Kang and Lee [18] found partial evidence that smoking causes depression. A recent systematic review of longitudinal studies regarding the associations between smoking and depression or anxiety examined the direction of the association. The results were inconsistent concerning the question whether smoking leads to depression or depression leads to smoking or whether, in addition, there is a bidirectional relationship between the two [50]. The authors also discussed that the association between smoking and depression may be the result of a shared genetic predisposition, an effect of environmental exposure (e.g., cigarette smoking) or other confounding factors (e.g., alcohol use) [50].

Extensive research has addressed the association between alcohol consumption and health effects, although the effects of alcohol consumption are controversial. In line with our results on late-life depression severity, several researchers point to a negative association between alcohol consumption and mortality in older adults especially when comparing the risk of moderate drinkers with that of abstainers and heavy drinkers [25,28,54]. It has been also discussed that the inverse association of moderate alcohol consumption is overestimated due to methodological biases [28,55,56]. However, older adults who drink moderately may be in better overall health, more social and outgoing and therefore more active; all factors that can potentially mitigate depressive symptoms [57]. In addition, moderate drinkers have been shown to have more favorable social and lifestyle characteristics compared to non-drinkers [58]. Occasional alcohol consumption may occur as part of shared social activity and is associated with more physical activity [31,59]. Physical activity in old age, in turn, has been shown to be associated with good overall health [60], while physical impairment is associated with an increased risk of depression [31,57]. However, reverse causality is also conceivable: those who are social drinkers might also drink, at least occasionally, in old age. The same is true for smoking: those who exhibit more severe depressive symptoms or had symptoms earlier in life might be more likely to remain smokers in old age. However, longitudinal analyses of all trajectories would be needed to investigate causal direction, leaving room for further investigation.

Other studies on alcohol use and mental illness have discussed a self-medication hypothesis that alcohol intake is used to attempt to improve mental and other health problems [20]. In particular, men appear to be more likely to use alcohol to cope with depression and other health problems than women [61]. Our results point in the same direction: only the male drinkers—but not the female—had a lower estimated severity of late-life depression on average, but only if they did not additionally smoke. It could be that older men have better well-being, are more active, healthier and more socially integrated. This could be because most of them (73%) were married and presumably well cared for. The women in our study were mostly widowed (60%), a factor associated with increased likelihood for depression [62]. Considering that older women consume less alcohol as self-medication, generally drink less alcohol, and consequently are less likely to drink socially, this could be a possible explanation for the lack of the (inverse) association between drinking and average depression severity.

After controlling for a large number of lifestyle and morbidity variables, earlier studies showed that the inverse association between moderate alcohol consumption and mortality remains consistent when considering other independent risk factors such as body mass index, health/functioning, physical health, social activities, and support of friends [24,28]. Nonetheless, the inverse association seems to be prone to factors like smoking and alcohol quantity [28]. This is consistent with our finding that men with concurrent substance use in particular are at increased risk for depression. Given that men tend to use both substances concurrently and in hazardous ways [29,30,31,61], particular attention in future studies should be paid to consumption patterns in order to identify high-risk groups.

### Strenghts and Limitations

The present study has several strengths. The multi-cohort study design provided a large sample of older individuals who are followed longitudinally. The inclusion of various original studies increases the external validity and generalizability of our results. Apart from this, there are some additional limitations. First, different measures of depressive symptomatology were used in the original cohort studies. Both scales are valid and commonly used. However, the use of variously designed instruments could raise questions about construct validity in the measurement of depressive symptoms. The CES-D is a rating scale that measures the intensity of depressed symptoms more precisely, while the GDS measures the frequency with a yes/no symptom list. To counteract this, we used a recognized procedure to harmonize data in terms of age, gender and educational differences. However, differences in mean severity of depression between the cohorts cannot be completely excluded, which should be considered when interpreting the results. In addition, potential biases may arise from inconsistencies in the reference period of drinking assessment. Second, the analyses are based on data from two cohort studies that show some heterogeneity in descriptive variables. For example, participants from the AgeCoDe/AgeQualiDe study are slightly younger, better educated, and somewhat more cognitively capable, while LEILA 75+ includes more very old women. This should be taken into account when interpreting the results. Nevertheless, we included LEILA 75+ because widowed very old women are a high-risk group for depression in old age [63], which is an important factor especially with regard to the examination of the association between depression and lifestyle. Third, we did not consider the dynamic of alcohol and tobacco use due to data limitations. Both variables were only assessed at baseline. Fourth, the survey is based on self-reported measures; therefore, influences such as social desirability cannot be excluded. Fifth, we did not control for prior drinking and smoking status, therefore a selection bias in observational data cannot be ruled out. Thus, for example, those with alcohol and tobacco problems might be less likely to enroll in cohorts at baseline due to poor health or simply early death [28,55]. Sixth, the current analysis did not control for other health, health-related, and sociodemographic variables because this data was not collected. This precluded a comprehensive examination of confounders and mediating factors, particularly for the association between alcohol and depression. Lastly, the focus of our study was on the association between depression severity and consumption variables, with effects particularly evident in men. However, it remains unclear how this could affect functioning in daily life, as well as quality of life in old age. Depressiveness is important, but only one aspect of functioning and quality of life. Another important aspect for the older and oldest old are physical limitations. Hence, future research may focus on how drinking and smoking affect physical functioning and activity.

## 5. Conclusions

In summary, associations suggesting a potential health benefit from moderate alcohol consumption should be interpreted with caution because of residual confounding factors and selection bias. In contrast, the positive association of smoking and depression severity appears to be well replicated. Furthermore, in this study, we showed that individuals who concurrently smoke and drink show higher levels of depression severity on average, regardless of gender. Our results have important public health implications. Because of the strong demographic trend toward an increase in life expectancy, accompanied by increasingly limited health-system resources, the maintenance of an independent and healthy lifestyle is more important than ever to meet the challenges of an aging population. Individuals, physicians and other stakeholders need well-founded scientific knowledge about the role of lifestyle factors such as drinking and smoking in promoting or worsening health in later life. This is even more true because these factors are potentially modifiable. Given the burden of major depression, it is important that health care providers, especially primary care physicians, continue to assess and monitor lifestyle factors in older age.

## Figures and Tables

**Table 1:** Sociodemographic characteristics at baseline for the total sample and by gender.

	Total Sample (N = 3724)	Gender		*p*-Value
		Women (*n* = 2469)	Men (*n* = 1255)	
Study, *n* (%)				<0.001
LEILA 75+	845 (22.7)	609 (24.7)	236 (18.8)	
AgeCoDe	2879 (77.3)	1860 (75.3)	1019 (81.2)	
Age, M (SD)	80.01 (3.9)	80.18 (3.9)	79.67 (3.8)	<0.001
Education, *n* (%)				<0.001
Low	2454 (65.9)	1697 (68.73)	757 (60.3)	
Medium	809 (21.7)	556 (22.52)	253 (20.2)	
High	461 (12.4)	216 (8.75)	245 (19.5)	
Marital status, *n* (%)				<0.001
Single/Divorced	465 (12.5)	394 (16.0)	71 (5.7)	
Married	1509 (40.5)	593 (24.0)	916 (73.0)	
Widowed	1750 (47.0)	1482 (60.0)	268 (21.3)	
MMSE, M (SD)	27.42 (1.9)	27.34 (1.9)	27.58 (1.8)	0.001

Note: M = mean, SD = standard deviation, MMSE = Mini-Mental-Status-Examination, *p*-values refer to significant testing of differences by gender.

**Table 2:** Substance use at baseline for the total sample and by gender.

	Total Sample (N = 3724)	Gender		*p*-Value
		Women (*n* = 2469)	Men (*n* = 1255)	
Drinking, *n* (%)				<0.001
No	1849 (49.6)	1527 (61.8)	322 (25.7)	
Yes	1875 (50.4)	942 (38.2)	933 (74.3)	
Smoking, *n* (%)				0.013
No	3472 (93.2)	2320 (94.0)	1152 (91.8)	
Yes	252 (6.8)	149 (6.0)	103 (8.2)	
Concurrent use, *n* (%)				<0.001
No	3584 (96.2)	2406 (97.5)	1178 (93.9)	
Yes	140 (3.8)	63 (2.5)	77 (6.1)	

Note: *p*-values refer to significant testing of differences by gender.

**Table 3:** Linear mixed-effects regression model with the harmonized depression scale (n = 3724).

Variable	Main Effects			Variable	Interaction Effects		
	Model 1				Model 2		
	Coef.	*p*-Value ^a^	95% CI ^b^		Coef.	*p*-Value ^a^	95% CI ^b^
Age at baseline	0.02	0.005 **	[0.012; 0.025]	Age at baseline	0.02	<0.001 ***	[0.012; 0.025]
Gender				Gender			
Women	Ref. ^c^			Women	Ref. ^c^		
Men	−0.116	<0.001 ***	[−0.175; −0.058]	Men	−0.12	<0.001 ***	[−0.175; −0.058]
Education ^d^				Education ^d^			
Low	Ref.			Low	Ref.		
Medium	−0.04	0.171	[−0.097; 0.017]	Medium	−0.04	0.165	[−0.097; 0.017]
High	−0.06	0.144	[−0.129; 0.019]	High	−0.06	0.144	[−0.129; 0.019]
Marital status				Marital status			
Single/Divorced	Ref.			Single/Divorced	Ref.		
Married, living in partnership	−0.03	0.446	[−0.113; 0.049]	Married, living in partnership	−0.03	0.451	[−0.113; 0.049]
Widowed	−0.07	0.073	[−0.142; 0.006]	Widowed	−0.07	0.074	[−0.142; 0.006]
Study				Study			
LEILA75+	Ref.			LEILA75+	Ref.		
AgeCoDe/AgeQualiDe	0.12	<0.001 ***	[0.068; 0.179]	AgeCoDe/AgeQualiDe	0.12	<0.001 ***	[0.068; 0.181]
MMSE ^e^	−0.04	<0.001 ***	[−0.049; −0.025]	MMSE ^e^	−0.04	<0.001 ***	[−0.049; −0.025]
Drinking				Drinking			
No	Ref.			No	Ref.		
Yes	−0.07	0.005 **	[−0.119; −0.021]	Yes	−0.08	0.001 ***	[−0.133; −0.032]
Smoking				Smoking			
No	Ref.			No	Ref.		
Yes	0.14	0.002 **	[0.051; 0.233]	Yes	0.03	0.625	[−0.103; 0.171]
				Drinking × Smoking	0.19	0.037 *	[0.011; 0.375]

Notes. ^a^ *p* = significance level; ^b^ CI = Confidence interval; ^c^ Ref.: Reference category; ^d^ educational classification according to the new CASMIN educational classification (Brauns & Steinmann, 1999); ^e^ MMSE = Mini-Mental-Status-Examination, *** significant on the level α = 0.001; ** significant on the level α = 0.01; * significant on the level α = 0.05.

**Table 4:** Linear mixed-effects regression with the harmonized depression scale stratified for gender.

Variable	Interaction Effects			Variable	Interaction Effects		
	Model 3 (Women *n* = 2469)				Model 4 (Men *n* = 1255)		
	Coef.	*p*-Value ^a^	95% CI ^b^		Coef.	*p*-Value ^a^	95% CI ^b^
Age at baseline	0.02	<0.001 ***	[0.010; 0.025]	Age at baseline	0.02	<0.001 ***	[0.009; 0.031]
Education ^d^				Education ^d^			
Low	Ref. ^c^			Low	Ref.		
Medium	−0.06	0.094	[−0.131; 0.010]	Medium	0.01	0.990	[−0.095; 0.096]
High	−0.07	0.210	[−0.169; 0.037]	High	−0.04	0.448	[−0.148; 0.065]
Marital status				Marital status			
Single/Divorced	Ref.			Single/Divorced	Ref.		
Married, living in partnership	−0.02	0.673	[−0.116; 0.075]	Married, living in partnership	−0.10	0.279	[−0.277; 0.080]
Widowed	−0.05	0.200	[−0.134; 0.027]	Widowed	−0.16	0.123	[−0.351; 0.042]
Study				Study			
LEILA75+	Ref.			LEILA75+	Ref.		
AgeCoDe/AgeQualiDe	0.10	0.004 **	[0.032; 0.169]	AgeCoDe/AgeQualiDe	0.17	<0.001 ***	[0.079; 0.269]
MMSE ^e^	−0.04	<0.001 ***	[−0.053; −0.023]	MMSE ^e^	−0.03	<0.002 **	[−0.056; −0.012]
Drinking				Drinking			
No	Ref.			No	Ref.		
Yes	−0.06	0.052	[−0.120; 0.001]	Yes	−0.14	0.004 **	[−0.231; −0.045]
Smoking				Smoking			
No	Ref.			No	Ref.		
Yes	0.08	0.321	[−0.079; 0.239]	Yes	0.13	0.335	[−0.395; 0.135]
Drinking × Smoking	0.13	0.301	[−0.113; 0.368]	Drinking × Smoking	0.39	0.013 *	[0.081; 0.695]

Notes. ^a^ *p* = significance level; ^b^ CI = Confidence interval; ^c^ Ref.: Reference category; ^d^ educational classification according to the new CASMIN educational classification (Brauns & Steinmann, 1999); ^e^ MMSE = Mini-Mental-Status-Examination, *** significant on the level α = 0.001; ** significant on the level α = 0.01; * significant on the level α = 0.05.

## Data Availability

The data presented in this study are available on request from the corresponding author. The data are not publicly available due to privacy and ethical reasons.
